# Strategic purchasing and health system efficiency: A comparison of two financing schemes in Thailand

**DOI:** 10.1371/journal.pone.0195179

**Published:** 2018-04-02

**Authors:** Walaiporn Patcharanarumol, Warisa Panichkriangkrai, Angkana Sommanuttaweechai, Kara Hanson, Yaowaluk Wanwong, Viroj Tangcharoensathien

**Affiliations:** 1 International Health Policy Program (IHPP), Ministry of Public Health, Nonthaburi, Thailand; 2 London School of Hygiene & Tropical Medicine, London, United Kingdom; University of Hawaii at Manoa, UNITED STATES

## Abstract

Strategic purchasing is an essential health financing function. This paper compares the strategic purchasing practices of Thailand’s two tax-financed health insurance schemes, the Universal Coverage Scheme (UCS) and the Civil Servant Medical Benefit Scheme (CSMBS), and identifies factors contributing to successful universal health coverage outcomes by analysing the relationships between the purchaser and government, providers and members. The study uses a cross-sectional mixed-methods design, including document review and interviews with 56 key informants. The Comptroller General Department (CGD) of Ministry of Finance manages CSMBS as one among civil servant welfare programmes. Their purchasing is passive. Fee for service payment for outpatient care has resulted in rapid cost escalation and overspending of their annual budget. In contrast, National Health Security Office (NHSO) manages purchasing for UCS, which undertakes a range of strategic purchasing actions, including applying closed ended provider payment, promoting primary healthcare’s gate keeping functions, exercising collective purchasing power and engaging views of members in decision making process. This difference in purchasing arrangements resulted in expenditure per CSMBS member being 4 times higher than UCS in 2014. The governance of the purchaser organization, the design of the purchasing arrangements including incentives and use of information, and the institutional capacities to implement purchasing functions are essential for effective strategic purchasing which can improve health system efficiency as a whole.

## Background

Purchasing is one among three key health financing functions [1], involving the transfer of pooled funds to healthcare providers to secure services for a population. Strategic purchasing has the potential to increase health system equity, efficiency and quality [2, 3]. A multi-country study of strategic purchasing arrangements was undertaken by the Resilient and Responsive Health Systems (RESYST) consortium to assess and identify areas for improvement in purchasing mechanisms in seven low and middle-income countries across Africa and Asia [4, 5].

Strategic purchasing involves three sets of decisions: (a) identifying the interventions or services to be purchased, taking into account population needs, national health priorities, cost-effectiveness and other factors; (b) choosing service providers, giving consideration to service quality, efficiency and equity and (c) determining how services will be purchased, including contractual arrangements and provider payment mechanisms [6]. In performing strategic purchasing, a clear regulatory framework and guidance by the government is required to ensure that public health priorities are linked to resource allocation and purchasing decisions. As the purchaser buys health services on behalf of the covered population, it is important for the purchaser to ensure that there are effective mechanisms in place to determine and reflect people’s needs, preferences and values in purchasing, and to hold health providers accountable to the people.

In 2002, Thailand achieved full population coverage through three public health insurance schemes. The Social Health Insurance (SHI) is a contributory scheme for private workers managed by the Social Security Office (SSO) of the Labour Ministry. The other two schemes are tax financed non-contributory schemes. The Civil Servant Medical Benefit Scheme (CSMBS) covers government employees and their dependents, and is managed by the Comptroller General Department (CGD) of the Ministry of Finance. The Universal Coverage Scheme (UCS) for the remaining population, who are not members of SHI or CSMBS, is managed by the National Health Security Office (NHSO).

Purchasing functions involve four key actors: the purchaser, the government, healthcare providers and citizens. By analysing the relationships between these actors, this study assesses and compares the purchasing functions of the UCS and the CSMBS. Lessons are drawn for low and middle income countries in their quest for progressive realization of Universal Health Coverage (UHC), in particular for efficiency improvement. This paper focuses on efficiency as it is easily measured by expenditure per member.

## Methods

A mixed method research design employing both quantitative and qualitative methods was applied. Secondary data were collected from CSMBS and UCS. The qualitative methods involved review of key documents and in-depth interviews and small group discussion with key informants.

Documents reviewed were mainly unpublished grey literature such as laws, regulations, governing board meeting minutes, annual reports of CSMBS and UCS; and published papers on the performance of the two schemes. Documents were retrieved from three sources. First, documents which were referred to by key informants, such as meeting minutes. Some unpublished documents were accessed through official requests from researchers. Second, we searched the websites of NHSO and CDG which focused on regulation and annual reports. Third, peer review published journals were earched from PubMed. The scope of data search covered the period since the inception of UCS in. S1 Table contains details of document reviews.

Key informants were drawn from three stakeholder groups. First, senior management of CGD and NHSO who are most knowledgeable about the policy and implementation of the two schemes; second, health care providers, reflecting their perspectives on working with CGD and NHSO; and finally beneficiaries from both schemes on their perspectives. In total 56 key informants were interviewed, including 13 senior managers from CGD and NHSO, 18 hospital managers and 25 UCS and CSMBS beneficiaries. Table 1 provides details of key informants’ profiles. Informed consent was obtained from each informant. Ethics approval was obtained by ethics bodies at the Institute for the Development of Human Research Protection, Thailand and the London School of Hygiene & Tropical Medicine.

**Table 1:** Profiles of key informants.

Key informant group	Male	Female	Total	Average age (min-max)	Average work experiences, year (min-max)
Purchasers: head of departments, four from CGD and nine from NHSO	6	7	13	50.7 (30–59)	12.8 (2–35)
Healthcare providers: director of hospital, head of insurance department, seven secondary and eleven tertiary hospitals	8	10	18	44.2 (33–58)	12.7 (4–33)
Beneficiaries, eight UCS and 17 CSMBS members	10	15	25	45.3 (29–58)	NA
Total key informants	24	32	56	46.3 (29–59)	12.8 (2–35)

Interviews and group discussions were recorded and transcribed in Thai. Data were analysed using content analysis according to a coding framework that was developed based on key purchasing functions and relationships between the two insurance agencies and the government, the healthcare providers and the beneficiaries [4].

## Findings

### CSMBS and UCS: Similarities and differences

Table 2 compares the profiles of the two schemes. Similarities are the dominant role of public healthcare providers and comprehensive benefit package; both schemes are managed by the public agencies (CGD and NHSO) which are financed by general tax through an annual budget allocation. Differences are the governance of the purchasers and the design of purchasing arrangements, including the provider payment mechanism. The CGD is a department of the Finance Ministry. The CGD Director General reports to the Finance Secretary in a bureaucratic line of accountability. In contrast, NHSO is an autonomous public agency established by the National Health Security Act 2002 with its own multi-stakeholder governing board—The National Health Security Board (NHSB).

**Table 2:** Comparison various dimensions related to purchasing functions of UCS and CSMBS.

	CSMBS	UCS
Purchaser organization	Comptroller General’s Department of the Ministry of Finance	National Health Security Office, an autonomous public agency established by law, with its own governing body.
What services are purchased?	Similar to UCS, but use of non-essential medicines is permitted if physicians confirm these are clinically indicated.	Comprehensive services, including medicines with reference to National List of Essential Medicines (NLEM) for UCS beneficiaries and Health promotion and disease prevention for all Thais (not only UCS members)
Who uses the services?	5 million members (8% of population) who are civil servants, government pensioners and their parents, spouses, three children less than 20 years old. Mostly live in urban and belong to rich quintiles.	The remaining 48 million, 75% of population who are not members of CSMBS and SHI; mostly rural population in the informal economic sector, 50% of them belong to the poorest and poor wealth quintiles
Who provides the services?	No Primary Health Care gate keeping, direct access to public hospitals and specialists. Access to private hospitals is only for life threatening accident and emergencies.	A contracting public primary care provider network, notably district health system, consisting health centres and a district hospital. The network serves gate keeping function. UCS members need to register with the District Health System (DHS) in their district of residence; UCS members do not have free access to providers outside their registered network unless they are referred. In urban areas, NHSO also contracts qualified private clinics to provide ambulatory care
How are providers paid?	Originally, fee-for-service was used for all services. Some beneficiaries had financial barrier of paying money upfront and getting reimbursement later. CSMBS reformed to disbursement from CDG directly to providers for out-patient services in 2003 and Diagnostic Related Group (DRG) for in-patient services in 2007. Currently, mixed provider payment applied which are (1) all outpatient services are paid on a fee for service basis, and directly disbursed from CGD to healthcare providers on a monthly basis; (2) inpatient services are paid by DRG without a global budget, different DRG base rates are applied, with a higher rate for teaching than district hospitals; and (3) other high cost interventions are paid by fee schedule, but at higher rates than UCS	Mixed provider payment applied which are (1) age adjusted capitation paid to district health system, based on the number of registered members in the catchment population. Costs of outpatient referral to higher level are the responsibility of the network; (2) hospitals are paid by DRG with national global budget. A single base rate per Relative Weight is applied to all levels of hospital and to both public and private facilities; (3) other high cost interventions such as dialysis, chemotherapy, antiretroviral treatment are paid on a fee schedule; and (4) health promotion and prevention for all Thais are mostly paid on a capitation basis with some combination of fee schedules.

**Source**: Authors’ synthesis

Note: Details of Social Health Insurance for private sector employees are not included in this table as it is outside the scope of this study.

**Fig 1:**
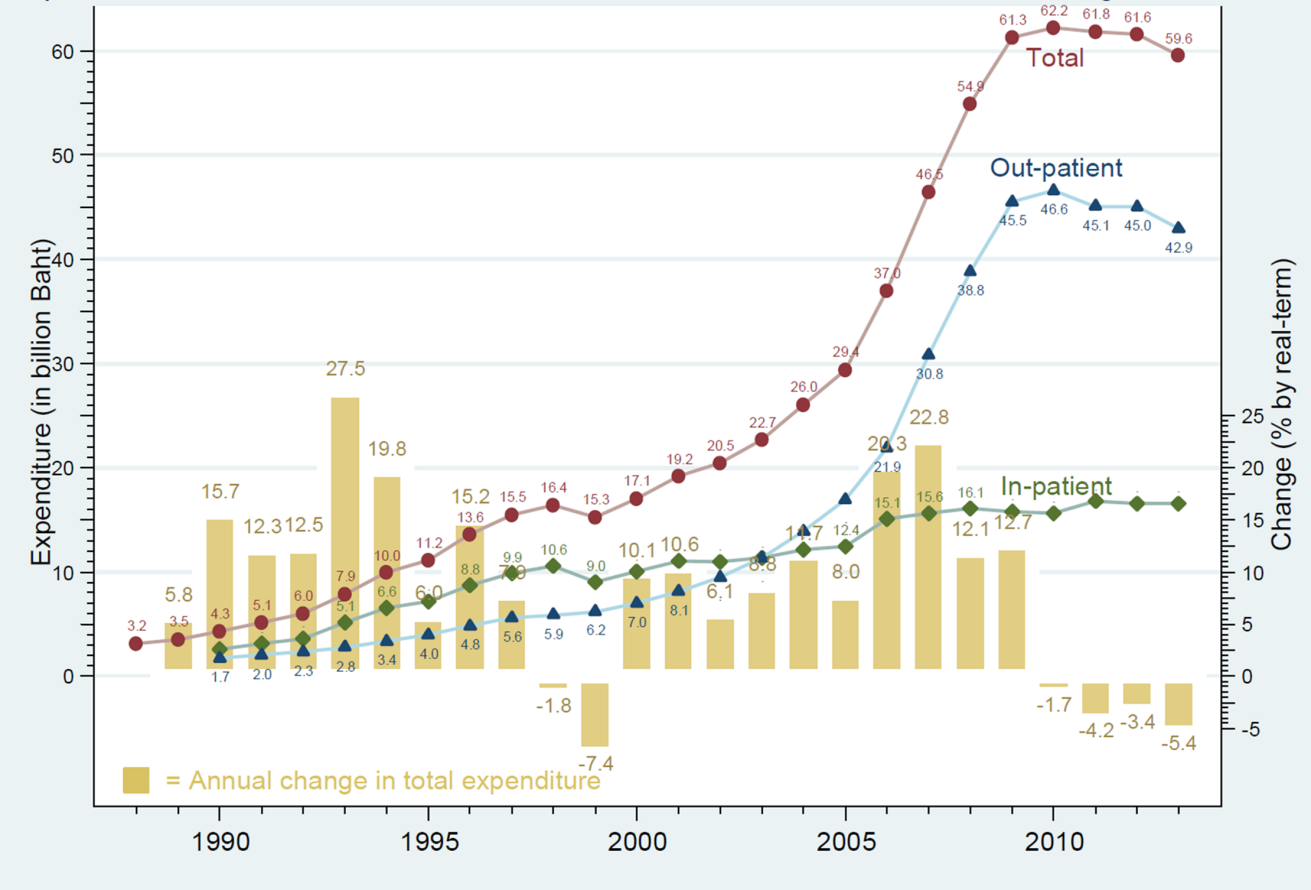
CSMBS annual expenditure: Total outpatient and inpatient care and annual growth 1988 to 2013.

Health expenditure per member of CSMBS was four times higher than UCS during 2012–2015 (Table 3). In 2015, CSMBS spent approximately 13,756 Baht per member (US$ 459, at 30 Baht exchange rate) while UCS spent 3,168 Baht per member (US$ 106). Outpatient services accounted for about 70% of CSMBS spending (Fig 1).

**Table 3:** Expenditure per member of CSMBS and UCS.

	2012	2013	2014	2015
**CSMBS**				
• Expenditure (mln baht) *	61,828	59,782	67,611	66,528
• Members **	4,967,575	4,878,258	4,837,927	4,836,208
• Baht / member ***	12,446	12,255	13,975	13,756
**USC**				
• Expenditure (mln baht) **	140,609	141,540	154,258	153,152
• Members **	48,620,104	48,612,007	48,312,428	48,336,321
• Baht / member ***	2,892	2,912	3,193	3,168
Differences of expenditure per member between CSMBS and UCS ***	**4.3**	**4.2**	**4.4**	**4.3**

**Source:** Authors’ compilation from several sources and calculation

* from Thai National Health Account

** Annual Report of UCS 2016

*** Authors’ calculation

Note that due to data limitation, members of CSMBS were mixed with number of state enterprises; so the health expenditure per member of CSMBS might be lower than it should be.

### CGD and NHSO: Relationship with the Government

#### Policy framework and governance: Determinants of accountability

There is neither a legal mandate nor self interpretation by CGD that it is a purchaser organization. The Decree of Ministry of Finance 1975 used the term "*Birk Jai”* meaning “public reimbursement” model for CSMBS which reflects that CGD has responsibility for financial transactions only.

“…The Law does not indicate the mandate of ‘purchasing’ for us. We have a mandate to reimburse money to the government officials. We have responsibility of controlling the reimbursement process according to the Ministry of Finance rules and regulations.” [Purchaser 3]

The National Health Security Act 2002 mandates NHSO to function as a purchaser agency for UCS. NHSB lays down the policy and strategic direction that the Secretary General of NHSO, appointed by the Board, has to follow and for which s/he is accountable through annual performance assessment. NHSB consists of 31 members from various stakeholder groups, including five representatives from NGO constituencies who represent the concerns of the UCS members in the Board’s decision making.

#### Budget and use of budget

Fee for service payment makes it impossible for the CGD to control the CSMBS budget, resulting in regular overspending of the annual budget allocation, with neither penalty nor challenge from the Ministry of Finance on CGD performance and efficiency. The shortfall is covered by re-allocation from other budget lines. CGD feels justified in using the government budget as these are entitlements of CSMBS members. There is no annual report on CSMBS performance.

“The medical benefit of CSMBS members is one of the welfare programs that the government provides to government officers in order to support and relieve them from the financial burden of sick people in his/her family.” [Purchaser 1]

In contrast, NHSO proposes and negotiates an annual budget envelope with the Bureau of Budget representing the Government, using a per capita approach, called the capitation budget. The capitation budget is arrived at by multiplying the capitation rate by the number of beneficiaries. The capitation rate is estimated based on use rates of different services such as outpatient and inpatient, and their unit costs. NHSO cannot and has never spent beyond the approved envelope.

#### Design of benefit packages and use of evidence

CSMBS does not have a clear mechanism for designing the benefit package, but relies solely on the expert opinion of their Technical Advisory Committee, made up of medical specialists. There is no management of conflict of interest in their advisory role.

Originally, the UCS applied the benefit package of the Social Health Insurance (SHI) scheme, which covered almost all interventions other than a few specified interventions such as cosmetic surgery and interventions of unproven effectiveness. Later, as capacity in Health Technology Assessment (HTA) was established in Thailand [7], new interventions were subjected to rigorous HTA [8]. One exceptional case is renal replacement therapy for renal failure patients; peritoneal dialysis and hemo-dialysis are not cost effective and the long term budget impact is substantial [9]. By assembling evidence on demand [10] and public opinion [11], in 2007, renal replacement therapy was included in the UCS benefit package [12], on the grounds of providing financial risk protection to households and ensuring equity across insurance schemes, as both CSMBS and SHI fully cover renal replacement therapy.

Another platform for encouraging efficient resource use is the Ministry of Public Health sub-committee on the National List of Essential Medicines (NLEM). This process also applies rigorous HTA when considering new medicines for inclusion in the NLEM which is referenced by both UCS and CSMBS to define medicine benefits [13].

NHSO has actively engaged in and supported HTA for new interventions and convenes a sub-committee under the NHSB to recommend which new interventions will be listed in the UCS benefit package [14].

### CGD and NHSO: Relationship with healthcare providers

#### Use of primary health care and gate keeping functions

There is no gatekeeping by primary care providers in the CSMBS, which provides its beneficiaries with a free choice of public hospital outpatient and specialist services. Gate keeping is further hampered by fact that primary health care, which has been well developed in all districts of the country [15], is not well developed in urban areas where the majority of CSMBS members reside. Interviews with CGD and beneficiaries found little support for gatekeeping, and that all respondents prefer having direct access to hospital and specialist services.

*“CSMBS allows us to get services from any government hospitals*. *It is impossible and not convenient at all for a registration to only one health center or one district hospital*. *We are working in a city; we should go to a government hospital nearby our offices…”* [Beneficiaries 1–12]

In UCS, the DHS, consisting of a district hospital and 10–15 sub-district health centres serving a typical catchment population of 50,000 people, carries out a gate keeping role. Patients who bypass the primary care level without a referral document need to pay medical bills out-of-pocket, which is not reimbursed by the UCS. This gate keeping function should lead to rational use of services by level of care with lower travel costs for patients.

#### Use of provider payment methods

**Fig 2:**
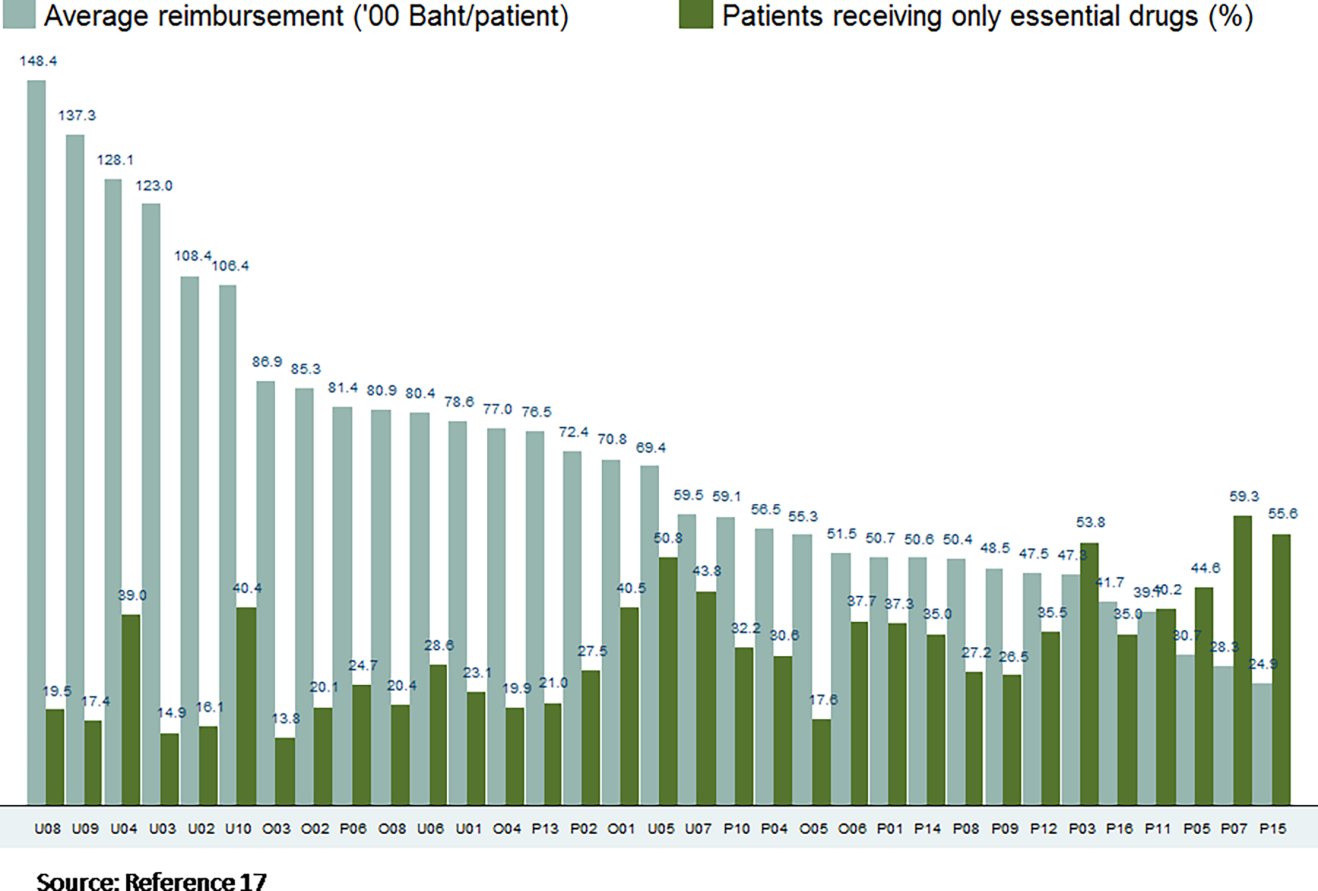
Average reimbursed drug expenditure and proportion use of essential drugs in 33 hospitals, July 2011 to March 2012.

CSMBS applies fee for service reimbursement for outpatient care, which encourages excessive use of medicines, especially those outside of the essential drug list. Among the 33 hospitals most visited by CSMBS members, hospitals with relatively higher expenditure prescribed a lower share of drugs from the NLEM. For example, University Hospital number 08 had the highest average drug reimbursement per patient at 14,840 Baht (US$ 495) per patient, and only 19.5% of total prescribed items were in the NEML (Fig 2) [16, 17].

In 2007 CSMBS replaced fee for service reimbursement for inpatient services by a multiple DRG reimbursement rate system, which pays higher base rates for tertiary and teaching hospitals, thereby encouraging their use. There is no discussion within the CSMBS of proceeding towards a single DRG rate for all hospital levels.

Fee for service, lack of gate keeping for primary care, free choice of tertiary teaching hospitals, and preferences for specialist care all result in excessive use of expensive outpatient services and cost escalation in the CSMBS. In 2009, CGD closely monitored 168 tertiary hospitals having more than 80% of total national outpatient caseload. A prescriber identification number for every clinician in these hospitals was introduced together with the use of electronic outpatient claims and application of a unique identification for each medicine to enable monitoring use of medicines by prescribers. This reform succeeded in halting the growth of total outpatient expenditure over the period 2010–2014, however, outpatient expenditure rebounded in 2015.

In contrast, the UCS uses closed-ended provider payment methods (capitation for outpatient care and DRG for inpatient care) and imposes restrictions on medicines to contain cost. The risk of DRG-creep (falsified up-coding of diagnosis in favour of higher DRG weight cases) is managed by applying a global budget on top of the DRG system and a thorough system of medical audit administered by the NHSO.

To avoid under-provision because of the closed-end payment systems, NHSO unbundled certain high cost interventions from the capitation and DRG payments and reimburses these on a fee schedule. It also uses central procurement for expensive medicines such as anti-retroviral treatment and peritoneal dialysis solutions.

#### Exercising collective purchasing power

Each government hospital has its own procurement mechanism for almost of all medicines, leading to highly atomised purchasing practices. There is a large potential cost saving and efficiency gain if CGD were to procure medicines centrally: one study among patients with high-cost medicines shows that cost of medicines was about 73% of total healthcare costs [18].

In contrast, NHSO uses its substantial purchasing power to negotiate lower prices for some selected high cost medicines and medical devices, leading to cost savings and a higher number of patients receiving medicines. In 2008, selected high cost medicines (classified as E2) are included in the NLEM with an aim of increasing access to high cost medicines for treating rare or complex conditions. The E2 lists initially cover 10 speciality medicines (e.g. docetaxel for breast and lung cancer) for 21 indications. The E2 program mandates all public insurance schemes to fully subsidize the E2 medicines when patients meet specific clinical criteria [19]. In January 2009, the NHSO implemented the E2 program by introducing central procurement with the Government Pharmaceutical Organization (GPO) for all E2 medicines used for UCS patients instead of individual hospital procurement. Healthcare providers are directly provided with these E2 medicines by GPO via a vendor-managed inventory system. The central purchasing implemented by NHSO and GPO in Jan 2009 resulted in lower prices of E2 medicines [18, 20] by approximately 25% (range 8–40%) [21] which allowed the number of patient receiving E2 medicines to increase significantly.

### CGD and NHSO: Relationship with their members

An external review [22] by international experts confirmed the performance of the UCS in terms of equitable access to and use of health services, low levels of unmet need [23] and financial risk protection while performance of CSMBS is limited.

#### Differences of CSMBS and UCS members

**Fig 3:**
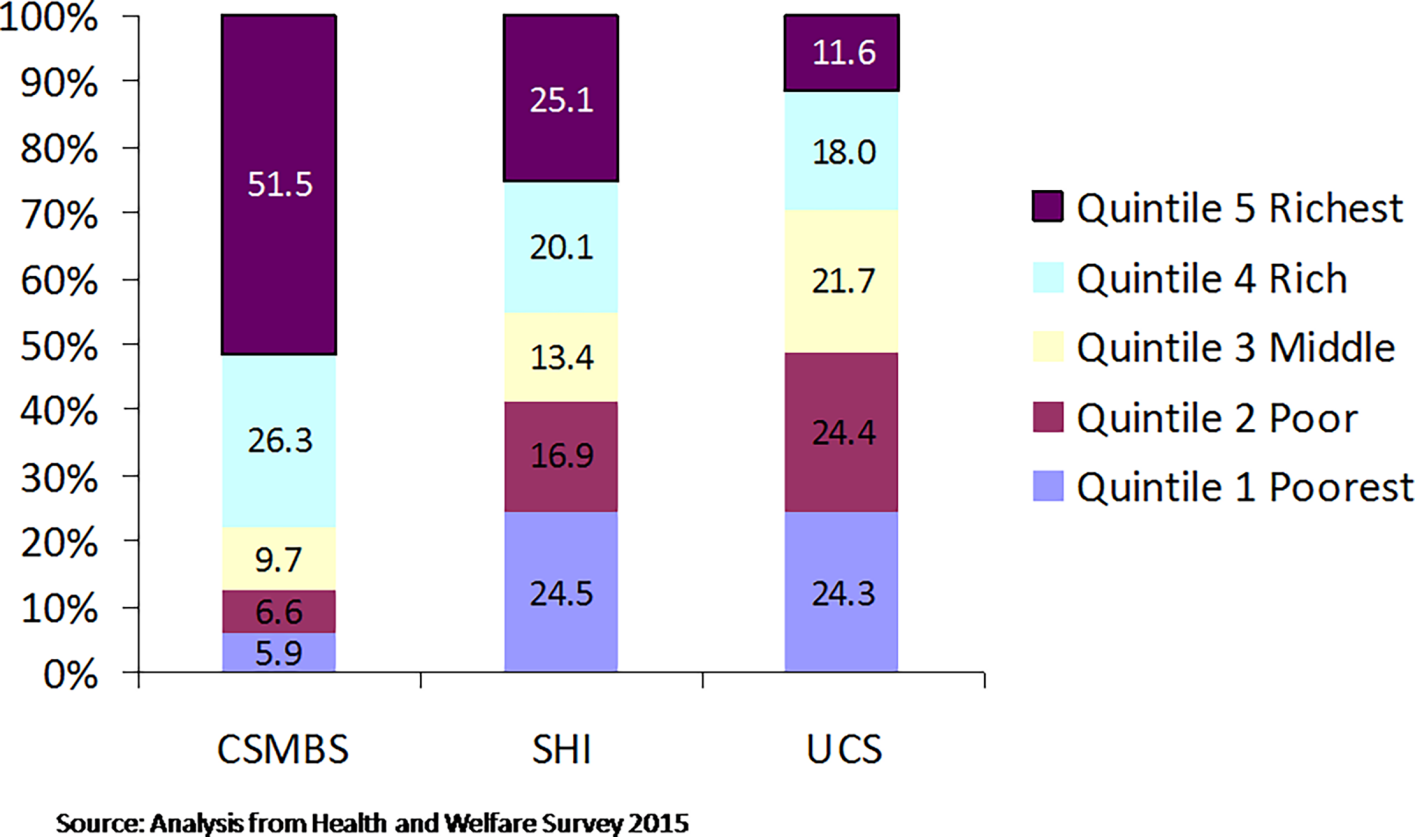
Economic status of beneficiaries across three public health insurance schemes.

CSMBS members reside in urban areas and are more educated and economically better off than UCS members (Fig 3) [24]. In order to verify member entitlements at the point of service use, the UCS uses a national electronic database with citizen ID numbers. This is facilitated by high coverage of civil registration of births (96.7%) and deaths (95.2%) [25]. Accurate data and real time updates are critical for UCS to allocate or re-allocate members and its capitation budget to a contracted DHS.

#### Members’ participation

The CGD call centre is available during government working hours which are inadequate to respond to the immediate problems of CSMBS members. There are 10,000 calls per year, or 180 calls per 100,000 members. These calls concern not only health but also other benefits. CSMBS does not have a regular forum for engaging with its members.

An annual public hearing of UCS members and healthcare providers is legally mandated and fully implemented by the NHSO. Gradually, the UCS has become owned by the people, not by the political party which initiated it in 2001. An NGO called “the UCS Fan” (‘PeopleHealthSystemsMovement’ on Facebook) was established some years ago. They closely monitor the actions of the government, the Ministry of Public Health and the NHSB. They advocate for an adequate annual government budget allocation for the UCS [26].

A 24-hour call centre service (known as “1330”), advises UCS members on entitlements and other service enquiries and manages disputes between patients and providers. There were 600,000 calls in 2014, or 1,280 calls per 100,000 UCS members (10 times higher than CSMBS) [27].

**Fig 4:**
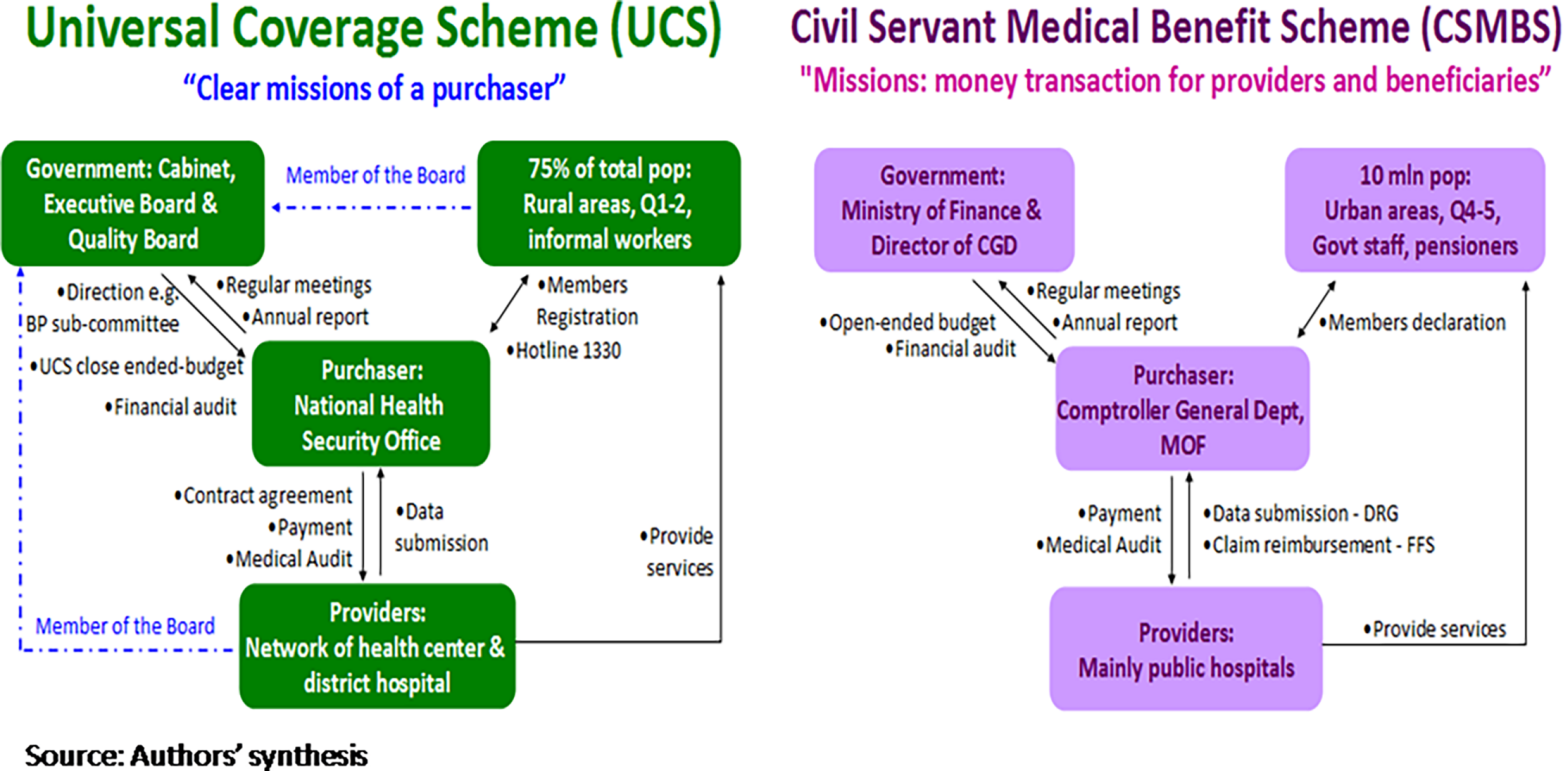
Summary of principal agent relationship between NHSO (left), CGD (right) and three main actors.

A summary of the relationships between the two schemes’ purchasers and their key stakeholders is illustrated in Fig 4.

### Factors influencing purchasing functions

Institutional capacity is critical for effectively undertaking the purchasing function. CSMBS is one out of sixteen mandates of the CGD, which is responsible for all other benefits and welfare systems. There is a total of 32 staff with no relevant background in health or public health. NHSO has the sole mandate of managing the UCS. It had 820 staff in 2016, divided among the central office and 13 regional NHSO offices. About one-third of total staff has a health background. Almost all the executive positions (67 out of 71 positions) are held by experienced and highly qualified medical and health professionals.

In addition, the multi-stakeholder NHSB of the UCS is an effective forum for ensuring accountability in decision making and represent the views of the taxpaying public and UCS members. A synthesis of factors which contribute to / undermine effective purchasing practices in the two schemes is summarized in Table 4.

**Table 4:** Contributing factors and undermining factors of purchasing functions.

	Contributing factors	Undermining factors
A. Legal framework	Clear policy, expectation and mandate of “a purchaser” for benefit of people and health system; NHSO has only one mandate of managing UCS.	Clear policy of not operating as a purchaser but merely a payer, responsible for financial transactions; CSMBS in one among sixteen mandates of CGD. Therefore CGD has many other important tasks including advice on financial management, public procurement in the organization.
B. Governing body, organization and accountability framework	An independent organization; Governing body of multi-stakeholders–citizens’ engagement; Adequate number and competency of staff (including many with health background).	Government structure with rigid mandate using command and control; Inadequate staff; Staff do not have health background.
C. Resource	Tight budget with tough process of negotiation resulting in careful management.	Soft budget constraint leads to inefficiency of the system.
D. Information	Information management; Using appropriate information, communication and technology; Pool of information from all contracted providers to the national level.	Fragmented data requirement of different schemes creates difficulties for providers.
E. Communication	Two way communication; Proactive communication–NHSO staff visited providers; NHSO conducts public hearing with citizens.	Official process of bureaucratic channels is ineffective.
F. Audit	Independent auditing mechanism; Team work of audit; An opportunity to improve knowledge and skill of the audit team and providers and to improve data quality of providers; Penalty and incentives must be implemented.	Negative attitude, perception and practice of investigators and being investigated persons.

Source: Authors’ synthesis

## Discussion

Together, the evidence of the expenditure per member and many activities of the two purchasers clearly show that UCS had more effective purchasing functions than CSMBS. These in turn are due to many factors, including an appropriate legal framework, organizational arrangements, and institutional capacity.

Strategic purchasing practices can improve health system efficiency. These include closed-ended provider payment methods, use of a NLEM and systematic HTA for new procedures, medicines and devices. The UCS has also avoided under-provision of services by unbundling certain high cost services from the capitation and DRG systems and replacing them with fee-for-service according to an established schedule. The exercise of purchasing power by the NHSO has reduced prices of key medicines and devices, and gate keeping of access to specialist care by the DHS promotes rational use of the referral system. This has been complemented by a well-functioning supply side, with extensive geographical coverage.

The problems of the CSMBS are clear and publicly known. Its inefficiency, cost escalation and greater per capita expenditure than that of UCS members, stem from the fee for service payment for outpatient service which triggers excessive use of non-essential medicines, while CGD has limited regulatory capacity to audit and a lack of commitment to reform the inherited weakness of fee for service. The lack of primary care provision, and provision of ambulatory services by the overcrowded outpatient departments of tertiary care hospitals are costly while quality of services is poor. Despite these challenges, solutions are not forthcoming. CGD has neither the commitment nor the technical capacity to introduce effective reforms. CGD’s efforts to prohibit the widespread use of ineffective glucosamine were determined to be unconstitutional by the Administrative Court after they were challenged by two CSMBS members [28]. There are conflicts of interest among medical professionals favouring glucosamine and the treatment guideline produced by Royal College of Orthopedists is unclear. CGD was criticized for attempting to outsource the management of CSMBS to private insurance agencies, as this measure would not address the fundamental problem of cost escalation caused by fee for service payment. CSMBS members are worried that their benefits will be cut [29].

The higher cost per capita CSMBS members was not due to a more generous benefit package than UCS. There are few differences in the benefit package between the two schemes. For example, dental services in CSMBS are slightly more extensive than UCS while UCS provides more health promotion than CSMBS. The CSMBS fee for service payment for outpatient results in prescription of more diagnostic procedures and medicines, which *de facto* provides higher service intensities although *de jure* there are no significant differences in the outpatient benefit package between the two schemes. The higher DRG payment rate paid by CSMBS for the same clinical conditions in tertiary and teaching hospitals results in higher cost to CSMBS.

Despite the successful outcomes of UCS, a few challenges remain. Its reliance on an annual government budget allocation runs the risk of lower budgets during the “lean years” of economic downturn and government fiscal constraint. Where the budget allocation doesn’t match the increased demand for health services, and labour and medicine cost inflation, implicit rationing and waiting lists may emerge. Policies to address these risks include a dedicated annual health tax, prepayment contribution with exemption of the vulnerable and poor populations and introducing copayment. To ensure equity across population groups, prepayment contribution and/or copayment must be applied to all three schemes.

## Conclusion

The study demonstrates that strategic purchasing can improve health system efficiency as a whole using multiple approaches i.e. using primary care as gatekeepers to promote appropriate use by level of care, use of a closed-ended budget with proper mix of provider payment methods, effective application of a NLEM, exercise of purchasing power, and strengthening civil registration for all schemes. These in turn are underpinned by a mix of design features, including legal framework, primary mandate, governance, provider payment mechanisms and institutional capacity of the purchaser.

Though they arise from a specific context, the lessons from comparing these two systems in Thailand should be useful for other countries considering how to strengthen strategic purchasing in the context of their UHC reforms.

## Supporting information

S1 Table Document review. (PDF)

## Abbreviations

CGD: Comptroller General Department, Ministry of Finance
CSMBS: Civil Servant Medical Benefit Scheme
DFID: UK Department for International Development
DHS: District health systems
DRG: Diagnostic Related Group
GPO: Government Pharmaceutical Organization
HTA: Health Technology Assessment
NHSB: National Health Security Board
NHSO: National Health Security Office
NLEM: National List of Essential Medicines
UCS: Universal Coverage Scheme
UHC: Universal Health Coverage
SHI: Social Health Insurance
SSO: Social Security Office
RESYST: Resilient and Responsive Health Systems
