# Supplementary material for: Strategic purchasing and health system efficiency: A comparison of two financing schemes in Thailand
Source: PLoS One. 2018 Apr 2;13(4):e0195179. doi: 10.1371/journal.pone.0195179 (PMC5880375; doi:10.1371/journal.pone.0195179)
Supplement: S1 Table — (PDF) [file pone.0195179.s001.pdf]

Supporting information Table S1

Document review
The nature of documents for reviews are mainly unpublished grey literatures such as laws, regulations, governing board meeting minutes, annual reports on CSMBS and UCS; and published papers on the performance of the two schemes. Documents are retrieved from three sources. First, documents which are referred to by key informants such as meeting minutes of the governing board of the two schemes. Some unpublished reports are accessed through official request by researchers. Second, we search the websites of NHSO and CDG which focused on regulation and annual reports. Third, peer review published journals are searched from PubMed. The scope of data search covers 2002 onward since the 2002 UCS inception. Please see a "supporting information file" containing details of document reviews.

Table S1 Document reviews of UCS and CSMBS

**Type of document**	**UCS**	**CSMBS**
A. Law and regulation	National Health Security Act 2002; Regulation health care provider registration 2004; Regulation of punishment of over claiming 2009; Regulation of public hearing regulation 2013; Guideline for assessment of Contractor Unit for Primary Care (CUP).	Thai Medical Benefit Royal Decree 2010; MOF guideline for medical expense disbursement 2010; Manual of medical audit 2010; Guideline of direct disbursement audit 2012; Direct disbursement of medical expense 2013; Guideline of using non-essential drugs 2013; Guideline of medical mark up 2013; Medical charge rate for public healthcare providers 2006; Guideline for IP direct disbursement for by DRG for private providers 2011; Medical charge rate: room and meals 2013; Guideline for medical expense disbursement of haemodialysis.
B. Meeting reports	NHSO Board meeting reports 2012-1014; Meeting report of medical record audit guideline development.	
C. Annual reports	NHSO annual reports 2003-2013; Report of financial and quality auditing 2013; Outcomes of UCS 2005-2012; Report of satisfaction of beneficiaries and providers 2003-2013; Report of satisfaction of beneficiaries by ABAC poll 2010.	Ministry of Finance annual report 2010 on Civil Servant Medical Benefit Scheme
D. Research/ Other evidence	Progress report of UCS to the Senate; NHSO contract for public and private providers; Life cycle of hospital registration; Health service and quality assurance; UC benefit package cost effectiveness analysis; Voice of the people in the Thai National Health Security System.	Thai Medicines Terminology for 9 target drugs groups version 1.0; Hospital categorization for CSMBS by DRG 2013; CSMBS performance assessment by Central office for Healthcare Information (CHI) 2013; Summary of CSMBS direct disbursement in by CHI 2013; Report of guideline to improve efficiency of CSMBS management by TDRI 2011; Proposed plan for CSMBS management and improvement by CHI 2012-2015; An analysis for drug cost containment under direct disbursement system for outpatients in CSMBS 2013; Report of policy analysis on elective surgeries in private hospitals 2013
E. Others	Power point presentation of NHSO staff in various events	
